# Visual Function and Neuropsychological Profile in Children with Cerebral Visual Impairment

**DOI:** 10.3390/children9060921

**Published:** 2022-06-19

**Authors:** Federica Morelli, Giorgia Aprile, Chiara Martolini, Elena Ballante, Lucrezia Olivier, Elisa Ercolino, Eleonora Perotto, Sabrina Signorini

**Affiliations:** 1 Developmental Neuro-Ophthalmology Unit, IRCCS Mondino Foundation, 27100 Pavia, Italy; giorgia.aprile01@universitadipavia.it (G.A.); martolini.chiara@gmail.com (C.M.); lucrezia.olivier@mondino.it (L.O.); elisa.ercolino@mondino.it (E.E.); eleonora.perotto@mondino.it (E.P.); sabrina.signorini@mondino.it (S.S.); 2 Department of Brain and Behavioural Sciences, University of Pavia, 27100 Pavia, Italy; 3 BioData Science Center, IRCCS Mondino Foundation, 27100 Pavia, Italy; elena.ballante01@universitadipavia.it; 4 Political and Social Sciences, University of Pavia, 27100 Pavia, Italy

**Keywords:** cerebral visual impairment, development, visual function, neuropsychological profile, functional vision, reading

## Abstract

Cerebral Visual Impairment (CVI) has become the leading cause of children’s visual impairment in developed countries. Since CVI may negatively affect neuropsychomotor development, an early diagnosis and characterization become fundamental to define effective habilitation approaches. To date, there is a lack of standardized diagnostic methods to assess CVI in children, and the role of visual functions in children’s neuropsychological profiles has been poorly investigated. In the present paper, we aim to describe the clinical and neuropsychological profiles and to investigate the possible effects of visual functions on neuropsychological performance of a cohort of children diagnosed with CVI. Fifty-one children with CVI were included in our retrospective analysis (inclusion criteria: verbal IQ > 70 in Wechsler scales; absence of significant ocular involvement). For each participant, we collected data on neuropsychological assessment (i.e., cognitive, cognitive visual, and learning abilities), basic visual functions (e.g., Best Corrected Visual Acuity—BCVA, contrast sensitivity, and ocular motor abilities) and global development features (e.g., neurological signs and motor development delay) based on standardized tests, according to patients’ ages. The results showed that oculomotor dysfunction involving saccades and smooth pursuit may be a core symptom of CVI and might have a significant impact on cognitive visual and other neuropsychological abilities. Furthermore, visual acuity and contrast sensitivity may influence cognitive, cognitive visual, and academic performances. Our findings suggest the importance of a comprehensive assessment of both visual and neuropsychological functions in children when CVI is suspected, which is needed to provide a more comprehensive functional profile and define the best habilitation strategy to sustain functional vision.

## 1. Introduction

Cerebral Visual Impairment (CVI) is defined as ‘a verifiable visual dysfunction, which cannot be attributed to disorders of the anterior visual pathways or any potentially co-occurring ocular impairment’ [1]. According to this assumption, CVI derives mainly from anatomical and/or functional anomalies of the retro-geniculate visual pathways, including optic radiations, occipital cortex, and visual associative areas [2]. A dysfunction in the oculomotor control system can also be present [3,4,5]. CVI has become the leading cause of visual impairment (VI) in developed countries [4,6], partly due to better treatment strategies for the peripheral causes of VI (e.g., retinopathy of prematurity, cataract, glaucoma) and increased survival of newborns with brain injuries [6,7]. Its effects may comprise perceptual, oculomotor, and cognitive visual dysfunctions, occurring in isolation or concurrent [8,9,10]. Although traditionally associated with pathologies causing early brain injury (e.g., periventricular leukomalacia or intraventricular hemorrhage in premature newborns, hypoxic-ischemic injury, Central Nervous System (CNS) infections, head trauma, neonatal hypoglycemia) [11,12], CVI has also been reported in other disorders, such as genetic syndromes (e.g., Williams syndrome, Turner syndrome) and neurodevelopmental disorders (e.g., autism spectrum disorders) [11,13,14,15,16]. In the last few years, there has been an improvement in the ability to diagnose CVI, with a further increase in the frequency of its reports [6].

There is considerable consensus on the necessity to identify CVI early, since its prompt diagnosis and characterization are fundamental to define the best treatment [11,14,17]. Previous works have pointed out the necessity of a classification of CVI subtypes based on visual function [1,18,19]. Providing an early CVI diagnosis still seems to be difficult, especially in toddlers and in the absence of associated low vision or neuromotor disorders [20]. Firstly, the heterogeneity of clinical manifestations, etiologies, and associated conditions and the lack of a multidisciplinary approach may lead to a delayed diagnosis [11,14,21]. Secondly, there is no international consensus on a standardized diagnostic assessment of CVI that takes into account children’s ages and developmental abilities [1]; the only shared recommendation is to adopt a multidisciplinary approach [3,22], and the different methods for CVI evaluation are chosen depending on the context [1,11,23]. Given the possible complexity of the clinical picture of CVI, a multidisciplinary and thorough assessment may reveal the visual, developmental, and cognitive profile of a child, providing information on how to individualize his/her habilitation [24]. Research in this field has proposed descriptions of the cognitive, neuropsychological, and cognitive visual profiles in children with periventricular leukomalacia (PVL), one of the most frequent conditions associated with CVI [12,25,26], as well as screening tools such as specific questionnaires [18,27] and assessment tools for perceptual and neuropsychological evaluation in this population [14].

This paper has as a first goal to describe the clinical and visual characteristics of a cohort of children affected by CVI, along with their neuropsychological profiles. The second aim is to evaluate whether basic visual functions (such as best corrected visual acuity and contrast sensitivity) and ocular motor functions (fixation, smooth pursuit, and saccades) influence the development of neuropsychological skills in children with CVI, considering a homogeneous subgroup within the same cohort. With these purposes, we considered neuropsychological (cognitive, cognitive visual, and learning abilities) parameters, taking into account that a cognitive visual deficit may be considered part of the CVI diagnosis. To date, few studies have been conducted to explore the relationship between visual and cognitive functions in children with CVI [24,28,29]. We believe that providing information on the effects of basic visual and ocular motor functions and development features on neuropsychological skills in CVI might help in (a) supporting a more accurate CVI characterization, and (b) exploring the impact of visual functions and development features on functional vision [30], which is strictly connected to everyday life and academic abilities such as reading. Furthermore, considering neuropsychological, basic visual, and ocular motor functions and development features together would draw a more comprehensive picture of CVI children’s functional profiles, allowing for the tailoring of habilitation interventions for school and social inclusion.

## 2. Materials and Methods

### 2.1. Patients

**Figure 1:**
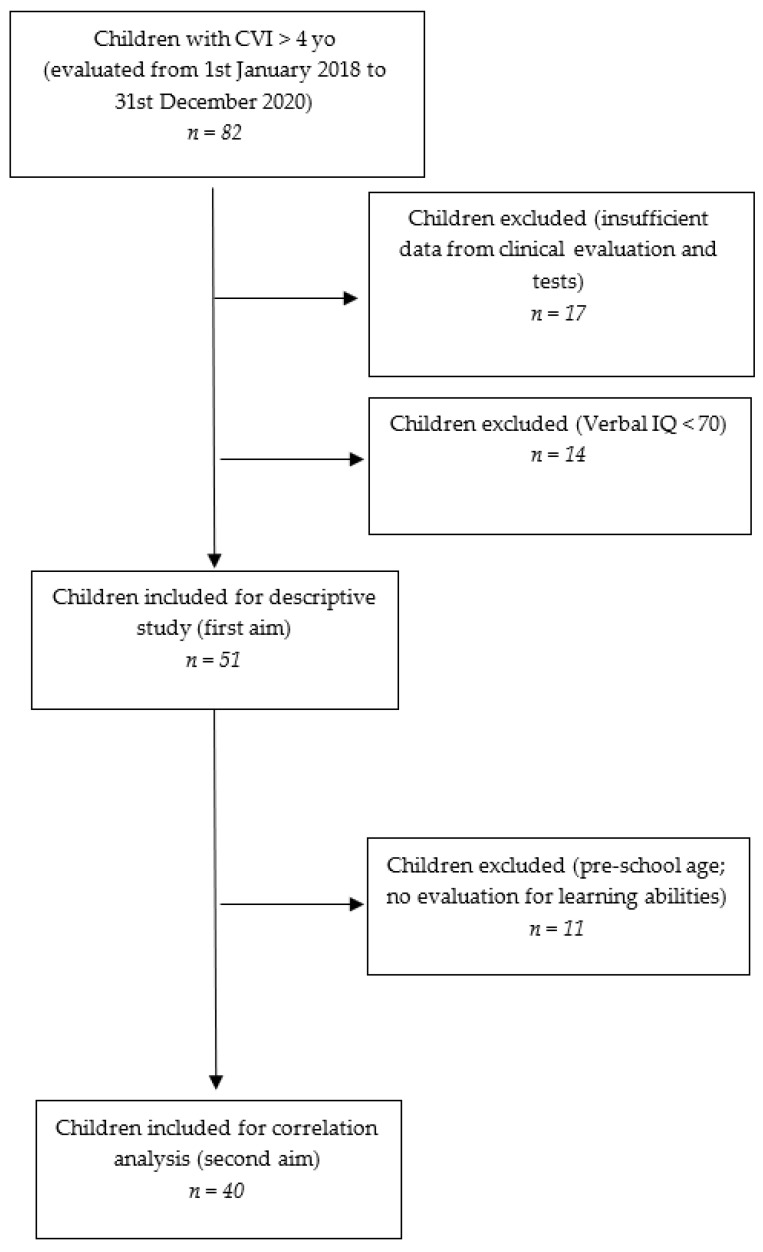
Flow chart of the retrospective cohort study.

We conducted a retrospective analysis on a cohort of pediatric patients referred to the Developmental Neuro-ophthalmology Unit of a tertiary referral hospital for neurological conditions (IRCCS Mondino Foundation, Pavia, Italy) from 1 January 2018 to 31 December 2020. CVI diagnosis was based on the definition reported in the Introduction [1]. As a clinical diagnosis, it relies on observations of children’s behavior (e.g., the pattern of peri-personal space exploration) and standardized evaluations of visual and visual cognitive functions, together with the support of diagnostic exams to exclude significant ocular involvement. For the diagnostic approach, we referred to previous works on the topic [2,20]. Medical history and diagnostic exams such as brain Magnetic Resonance Imaging (MRI) and Visual Evoked Potentials (VEP) suggesting CNS abnormalities further supported the diagnosis. Data concerning clinical details, neuro-ophthalmological evaluations, and neuropsychological test batteries of 82 children affected by CVI from different etiologies and aged above 4 years old (an adequate age to perform a more comprehensive neuropsychological and visuo-cognitive evaluation) were retrospectively collected. All the evaluations were chosen based on the ages and clinical pictures and performed for clinical purposes by a multidisciplinary team of professionals including child neuropsychiatrists, ophthalmologists, psychomotor therapists, and neuropsychologists with expertise in the field. Inclusion criteria were (1) a diagnosis of CVI; (2) a normal verbal IQ (>70) on Wechsler scales as considered in previous studies on similar topics [25]. Exclusion criteria were established as follows: (1) missing data for most of the clinical evaluations and tests (17 children), (2) Verbal IQ < 70 on Wechsler scales (14 children), (3) presence of a peripheral VI (i.e., caused by such conditions as retinopathy of prematurity or retinal dystrophy, which can directly affect visual perception) (no child met this criterion). A total of 31 children met the exclusion criteria, and 51, with a mean age of 113.07 months (range 62–213) ± 35.7, were preliminarily included (Figure 1) for the general cohort description study. Afterwards, a subgroup of 40 patients, homogeneous in terms of age and performed tests (mean age 121 months, range 78–187, ±29.3) was selected to investigate possible correlations between general clinical and visual features and neuropsychological skills. Subjects included for the correlation analyses were all primary or middle school children, to reduce the age range and to provide more homogeneity in cognitive tests (all children performed WISC-IV scales, and the majority of them were tested for learning abilities).

Being a retrospective analysis on data originally collected for clinical purposes, Ethics Committee approval was not required.

### 2.2. Procedure

The charts’ review focused on clinical history, neurological examination, and brain MRI (see Table 1 for details).

**Table 1:** General characteristics of the sample. GMFCS: Gross Motor Function Classification System. PVL: Periventricular Leukomalacia. IVH: Intraventricular Hemorrhage. CNS: Central Nervous System. N = 51.

**Parameter**	**Category**	**N (%)**
**Sex**	Male	25 (49)
	Female	26 (51)
**Mean age (months)**	113.07 (range 62–213) ± 35.7	
**Gestational age**	Term	4 (8)
	Late preterm (34–36 weeks)	15 (29)
	Moderate preterm (32–34 weeks)	3 (6)
	Very preterm (28–32 weeks)	8 (16)
	Extremely preterm (<28 weeks)	17 (33)
	Unknown	4 (8)
**GMFCS**	Level I	14 (27)
	Level II	18 (35)
	Level III	15 (29)
	Level IV	3 (6)
	Level V	1 (2)
Neuroradiological findings **	PVL (mild/severe)	33 (65)
	Sequelae of IVH or periventricular haemorrhagic Infarction	5 (10)
	Combination of PVL and IVH sequelae	1 (2)
	Basal ganglia/thalamus lesions (mild/moderate/severe)	1 (2)
	Cortico-subcortical lesions only (watershed lesions in parasagittal distribution/multicystic encephalomalacia) not covered under C3	1 (2)
	Arterial infarctions (middle cerebral artery/other)	2 (4)
	Miscellaneous	2 (4)
	Normal	1 (2)
	Unknown	5 (10)
**Neurological Picture**	Unilateral cerebral palsy	14 (27) *
	Bilateral cerebral palsy	30 (59) *
	Early CNS injury w/out neuromotor deficit	7 (14)
**Neurologic comorbidity (epileptic abnormalities)**	Not reported	44 (86)
	Reported	7 (14)
**Psychiatric comorbidity (anxiety, hyperactivity)**	Not reported	43 (84)
	Reported	8 (16)
**Neurological signs**	Diplegia	21 (41)
	Hemiplegia	15 (29)
	Tetraplegia	8 (16)
	Motor incoordination	4 (8)
	None	3 (6)
**Motor delay**	Unknown	6 (12)
	Not reported	14 (27)
	Reported	31 (61)
**Language delay**	Unknown	3 (6)
	Not reported	36 (71)
	Reported	12 (24)
**Type of therapy**	No habilitation	5 (10)
	Only physical therapy	24 (52)
	Physical and psychomotor	7 (15)
	Physical and speech therapy	3 (7)
	Physical, psychomotor, and speech therapy	2 (4)
	Psychomotor only	7 (15)
	Speech only	2 (4)
	Psychomotor and speech	1 (2)

* All patients with cerebral palsy (CP) had a spastic form, except for one patient, who had a dyskinetic bilateral CP. ** according to Himmelmann et al. classification system [31].

When referred to our Center, all children underwent an evaluation protocol comprising basic visual functions (such as visual acuity for far and near distances and contrast sensitivity), ocular motor abilities, and neuropsychological competencies, according to a protocol derived from the Center professionals’ experience and including cognitive visual aspects, as in Fazzi et al. [2,25,32]. Basic visual functions, ocular motor abilities, and neuropsychological assessments were performed by trained professionals (child neuropsychiatrists, therapists, psychologists, and orthoptists) with expertise in the diagnosis and habilitation of visual disorders. All the subjects also underwent an ophthalmological evaluation performed by an ophthalmologist with neuro-ophthalmologic expertise.

Concerning visual functions, we retrospectively considered the following parameters, according to a protocol presented by Fazzi et al. [2], categorized as exposed in Table 2:

- Best Corrected Visual Acuity (BCVA). All children in the sample were above 4 years of age (mean age: 113.07 months, SD: ±35.7; age range: 62–213 months), and their visual acuity was assessed using line tests (symbolic or literal optotypes, according to their age), both for near (40 cm) and far (3 m) distances. Recognition acuity was measured with the Snellen chart [33] or LEA vision test [34].
- Contrast Sensitivity (CS): the ability to detect an image’s photometric contrast and spatial frequency, evaluated with the LEA low contrast symbols test or Hiding Heidi [35], based on the age and level of cooperation of the patient.
- Fixation (F), indicated as the ability to maintain fixation on a target.
- Smooth Pursuit (SP), indicated as the ability to follow the trajectory of a slow-moving object both on a horizontal and vertical arc.
- Saccades (SC), indicated as rapid re-fixation eye movements.
- Extrinsic Ocular Motility (OM) indicated as extraocular movements.

The neuropsychological assessment included (see Table 3, Table 4 and Table 5):

- Cognitive assessment, with the following tests:
  ▪ The Wechsler Preschool and Primary Scale of Intelligence (WPPSI-III) [36] or Wechsler Intelligence Scale for Children (WISC-IV) [37] were performed according to the age of the child. For the WISC-IV scale, we collected the following scores: (i) Verbal Comprehension Index (VCI); (ii) Perceptual Reasoning Index (PRI); (iii) Working Memory Index (WMI); (iv) Processing Speed Index (PSI); (v) (TIQ). For WPPSI-III scale, we collected the following scores: (i) Verbal Comprehension Index (VCI); (ii) Performance Index (PI); (iii) Processing Speed Index (PSI); (iv) Total Intelligence Quotient (TIQ); (v) General Language Index (GLI). Additionally, we included the weighted scores derived from each subtest.
- Cognitive visual assessment, with the following parameters:
  ▪ The Developmental Test of Visual-Motor Integration (VMI) [38], performed along with its subtests, i.e., Visual Perception (VMI-V) and Motor Coordination (VMI-M), expressed in terms of percentile scores and categorized into normal (>16°P), frailty (5°–16°P), and deficient (<5°P);
  ▪ The Developmental Test for Visual Perception (DTVP) [39]: General Visual-Perceptual (DTVP-GVP), Non-Motor Visual-Perceptual (DTVP-NMVP), and Visual-Motor Integration (DTVP-VMI) quotients were collected and categorized as normal (>16°P), frail (5°–16°P) and deficient (<5°P).
- Learning abilities, with the following parameters, categorized as normal or deficient based on the Z-score:
  ▪ The Battery for Dyslexia and Developmental Dysorthography (DDE-2) [40], which is a commonly used Italian battery for the assessment of dyslexia and dysorthography. Specifically, the battery evaluates the ability to read and write both meaningful (DDE-MF) and non-meaningful (DDE-NMF) words by taking into account speed (VEL) and accuracy (ERR);
  ▪ The MT-3 test [41], which is a currently used Italian instrument aimed to evaluate comprehension (MT-COMP), reading accuracy (MT-RCOR), and reading speed (MT-RVEL) by proposing tests appropriate to the patient’s level of education.

### 2.3. Data Analysis and Statistics

Data were analyzed by using the free software R Version 4.1.2 (Free Software Foundation, Boston, MA, USA). To evaluate whether our sample had sufficient power to compute a statistical analysis on each dependent variable (i.e., cognitive, cognitive visual, learning), we computed the power analysis by calculating the sample size on the free software G*Power 3.1 (Faul et al., 2009 [42]), based on the following parameters (see Sakki et al., 2021 [19]):

− Effect size d_z_: 1.20;
− α err. prob. = 0.05;
− Power (1-β err. prob.) = 0.95.

The calculated sample size was = 10. Consequently, we excluded neuropsychological variables with a total sample smaller than 10 subjects (see Table 3, Table 4 and Table 5 in the main text for further details), i.e., The Wechsler Preschool and Primary Scale of Intelligence (WPPSI-III). In our sample, the variable with the higher number of missing values had 29 observations.

The numerical variables (related to cognitive assessment) do not present deviation from the normal distribution (*p* values of the Shapiro-Wilk test > 0.1 and visual inspection of qqplots; histograms are shown in the Figure S1 in the **Supplementary Materials**), so parametric models are adopted. We ran linear models separately on each part of cognitive assessment (WISC-VCI, WISC-PRI, WISC-WMI, WISC-PSI, WISC-IQ), considered as dependent variables. Each model considers the set of visual functions as covariates (BCVA, visual acuity for near distance, fixation, pursuit, saccades, ocular motility, contrast sensitivity). We ran ordinal regressions to investigate the influence of the same set of covariates on visuo-cognitive parameters (VMI, VMI-V, VMI-M, DTVP-GVP, DTVP- NMVP, DTVP-VMI, which represent target variables for each model). Finally, we ran logistic regressions to investigate the influence of the same covariates on learning abilities (DDE-MF-VEL, DDE-MF-ERR, Dysorthography, DDE-NMF-VEL, DDE-NMF-ERR, MT-RVEL, MT-RCOR, MT-COMP, which represent target variables for each model).

## 3. Results

In the present work, we evaluated the most relevant effects of basic visual functions, ocular motor abilities, and global development features on neuropsychological (including cognitive visual) performances. Results concerning the descriptive data for the cohort are reported in Table 1 and Table 2. Concerning statistical analyses, we report the significant results obtained. Other results are exposed in **Supplementary Materials** (Tables S1 and S2).

Concerning basic visual functions, BCVA for distance was shown to be negatively correlated with the WISC-IV scale’s Perceptual Reasoning Index (PRI, *p* value = 0.001) and Processing Speed Index (PSI, *p* value = 0.007), with worse performances in children with worse visual acuity. The same trend was found for the Developmental Test of Visual-Motor Integration in its global (VMI, *p* value = 0.03) and Visual Perception (VMI-V; *p* value = 0.006) scores. On the contrary, BCVA for near showed an opposite influence on VMI and VMI-V (*p* value = 0.03 for both), with worse performances in children with better visual acuity.

An altered Contrast Sensitivity was found to negatively influence the WISC-IV scale’s Working Memory Index (WMI, *p* value = 0.01), along with the Total Intelligence Quotient (TIQ, *p* value = 0.04). Another interesting result concerning the impact of contrast sensitivity was obtained from applying linear regression analysis to learning abilities: an altered contrast sensitivity showed a negative impact on a subtest concerning text comprehension (MT-text Comprehension—MT-COMP, *p* value = 0.007), and a borderline significance was also found regarding the DDE-2 Meaningful Words reading speed (DDE-MF-VEL, *p* value = 0.08).

Concerning ocular motor abilities, a more qualitatively durable and/or complete Smooth Pursuit was found to positively influence the WISC-IV scale’s Processing Speed Index (*p* value = 0.02) and all the VMI subscores (VMI *p* value = 0.04; VMI-V *p* value = 0.01; VMI-M *p* value = 0.03).

Furthermore, the Visual-Motor Integration subtest of the Developmental Test for Visual Perception (DTVP-VMI) was found to be positively influenced by a qualitatively better Extrinsic Ocular Motility (*p* value = 0.02), and better organized Saccades (*p* value = 0.04).

For further details on the statistical analyses results, see Tables S1 and S2 in **Supplementary Materials**.

## 4. Discussion

In the present paper, we describe the clinical, visual, and neuropsychological profile of a cohort of 51 children diagnosed with CVI. Furthermore, we investigate the possible influence of basic visual functions (e.g., Best Corrected Visual Acuity—BCVA, contrast sensitivity—CS) and ocular motor abilities (fixation, smooth pursuit, saccades, and extrinsic ocular motility) on the neuropsychological profile (i.e., cognitive, cognitive visual, and learning abilities) in a subgroup of children from the same cohort. Our aims are in line with the current literature on habilitation [43] in children, which encourages an approach based on the individual’s functional profile. In this view, an early and comprehensive evaluation approach to every child with diagnosed or suspected CVI would be helpful to tailor their habilitation program.

### 4.1. Clinical, Visual Function and Neuropsychological Profiles

Most of the subjects in our cohort (43/51, 84%) were born prematurely and 86% were diagnosed with Cerebral Palsy (CP). Moreover, the majority of them showed Periventricular Leukomalacia (PVL), sequelae of Intraventricular Hemorrhage (IVH) or periventricular hemorrhagic infarction in brain MRI (see Table 1 for further details), findings frequently associated with premature birth [31,44]. These data are in accordance with the current literature on CVI causes and associated conditions. Perinatal problems due to premature birth are considered the most common reason for acquired CVI [45,46], and prematurity is frequently also reported in cases of CVI with multiple etiologies [47]. Even though an etiological and radiological analysis goes beyond the purpose of this paper, these results may have interesting implications for the clinical management and follow-up of these children. Indeed, ‘at risk’ children who are already under clinical follow-up would benefit from early CVI screening, requiring attention to medical history and clinical characteristics such as VI in absence of an ocular problem of such entity to justify a functional deficit [1,4,10,17]. Children above 3 years of age would also benefit from screening tools such as specific questionnaires [10,18].

Concerning visual function profiles, CS and BCVA evaluated for far and near distance in our sample and were mainly within the normal or near-normal range. A slightly bigger percentage of children in the ‘mild low vision’ group was found for near distance, which could be interpreted on the basis of a foveal crowding phenomenon impairing symbol recognition in linear optotypes [48]. Although low vision and altered CS are common signs of CVI [2,49], good values of BCVA in CVI have also been reported [50,51]. Furthermore, some authors have described an improvement of BCVA during infancy in the absence of ophthalmologic or neurologic comorbidities (e.g., epilepsy) [47,52]. These could be possible explanations for the presence of normal or near-normal BCVA values in our sample, in which the mean age at evaluation was 9 years old and no child suffered from significant neurologic comorbidities such as epilepsy. Nonetheless, our findings should be interpreted with caution because of the retrospective nature of this paper and the restrictive inclusion criteria. In fact, only children with sufficient BCVA to perform neuropsychological assessment were included. On the other hand, it is worth highlighting that clinically tested visual abilities might not represent visual functioning in everyday life [22,53]. Indeed, as explained in a valuable work by Colenbrander [30], visual function performances (e.g., *‘how the eye functions’*) should not be considered without an appropriate *functional* visual behavior assessment (e.g., ‘*how the person functions*’), which should consider factors such as the subject’s environmental and social context. Many authors state that the most frequent symptoms observed in children with normal VA are visual perception and integration dysfunctions, due to damage to the associative areas (dorsal and ventral streams) [54], and ocular motor abnormalities [2,55]. Accordingly, visual function profiles in our cohort were dominated by abnormalities in ocular motor functions (i.e., smooth pursuit and saccades). An impairment of such functions in patients with CVI has been widely investigated [8,56,57] and may reflect dysfunctions in the oculomotor system and/or in the dorsal stream pathway involved in ocular movements and visually guided actions [5,8,56,58,59,60]. Nevertheless, most previous studies have characterized oculomotor dysfunction in children with CVI, mainly focusing on the presence of strabismus and/or nystagmus [26,32,61] or on the description of possible visual disorders in CP [2,5]. To our knowledge, only a few clinical and qualitative characterizations of these features have been performed [2,5]. Among quantitative studies, Newsham et al. [62] examined saccades and smooth pursuit in a group of very preterm children without major CNS involvement, revealing a modest latency in pursuit, while Jacobson et al. [63] reported altered smooth pursuit in children with PVL. An interesting prospective study by Kaul et al. [64] found significant correlations between ‘gaze gain’ (i.e., a combination of visual tracking through smooth pursuit, head movements, and saccades), quantitatively evaluated with electro-oculography at the age of 4 months, and later cognitive, language, and fine motor development, evaluated with the Bayley Scales for Infant Development at 3 years of age.

Concerning neuropsychological aspects, the total IQ mean value was in the borderline range (we specify that a normal verbal IQ was an inclusion criterion). The majority of children who performed the relative tests showed impairments in cognitive visual performances, manifesting themselves as frailties or as frank deficits, and several children had difficulties in object recognition tasks; these results confirm the notion that cognitive visual deficits may be a core symptom of CVI [11,21]. Even in a relatively small sample, our results seem to confirm the ones in a recent work from Ben Itzhak et al. [18,24] concluding that deficits in ‘ventral’ tasks such as object recognition might be a specific characteristic in children with CVI [24]. Concerning reading abilities evaluated with tests standardized for the Italian population, our sample showed an involvement of reading speed, with minor compromise of comprehension and accuracy. Such a finding could be related to visual characteristics, such as oculomotor impairment and the crowding effect, but also to attention, a function that is frequently altered in premature children [65,66].

### 4.2. Relation between Visual Functions and Neuropsychological Profile

In the second part of our work, we aimed to evaluate whether basic visual functions and ocular motor abilities would impact on neuropsychological performances in a group of 40 children with CVI.

Concerning basic visual functions, we found that children with normal BCVA values (>7/10) performed significantly better both in the Perceptual Reasoning Index (PRI) and in the Processing Speed (PSI) of the Wechsler intelligence scales. In addition, the Developmental Test of Visual-Motor Integration (VMI), in its global and visual perceptual (VMI-V) performances, appeared to be influenced by visual acuity for far distance (*p* = 0.03 and *p* = 0.006, respectively). This finding would suggest that an optimal level of visual experience is important for perceptual and abstract visual tasks, especially when characterized by high visual involvement and requiring visual–motor coordination. On the contrary, higher levels of visual acuity for near distance seem to negatively influence both global VMI and VMI-V performances. Such an unexpected result could be explained with the sample characteristics (the near-distance visual acuity was in the normal or near-normal range for 33/40 subjects, and only 3 subjects presented moderate low vision), in which severely visually impaired children were not present. In addition, from a theoretical perspective, we know that visual acuity alone is not sufficient to explain the performance in visuo-cognitive tasks requiring visuo-spatial abilities, which are influenced by the involvement of non primary-perceptive cerebral areas such as the dorsal stream [2,55].

The influence of BCVA on cognitive and visuo-cognitive tasks would argue in favor of a multidisciplinary approach, both in the evaluation and in the re-habilitation settings, in children with CVI. On one hand, visual acuity evaluation should always be considered when interpreting a child’s cognitive performance, especially when the cognitive profile is uneven with worse performances in processing speed, perceptual reasoning, and visual–motor coordination. On the other hand, sustaining visual acuity from an early age (for example, with multisensory activities) might be important to promote the integration of vision, implement perceptual development [67], and finally reduce the frailties that may already exist in such tasks due to the involvement of associative areas [2,55].

Another interesting finding concerning basic visual functions is the influence of contrast sensitivity (CS) on reading tasks. Specifically, CS seems to influence text comprehension tasks with a strong level of significance (*p* = 0.007) and word reading speed, though without reaching statistical significance (*p* = 0.08). Such a finding may have significant implications in light of the above-mentioned importance of environmental adaptations to sustain both visual function and functional vision in children [30,43]. Indeed, adapting school environment and material, for example, providing adequate illumination and high-contrast sheets, would sustain and improve the child’s reading performance.

Concerning ocular motor abilities, we found a significant influence on the same neuropsychological tasks requiring good visual–motor and visual perceptive abilities described for basic visual functions. In fact, a discontinuous smooth pursuit and altered saccades seem to affect all the components (visual and motor) of the Visual Motor Integration tasks. These results are in line with the hypothesis of Kaul et al., who postulated that higher visual functions (evaluated, for example, with visual motor integration and perceptual reasoning tasks) may be influenced by oculomotor abilities in premature children [64], and would argue in favor of early oculomotor training to sustain systematic visual exploration.

To our knowledge, this is one of the first works investigating a role of CVI in school performances. Literature suggests that deficits in functions such as attention, ocular motility, and visuospatial processing (which are frequently associated with CVI) may have a repercussion on academic performance, which is considered an aspect of *functional vision* [30,68,69]. In our study we found promising results regarding the influence of contrast sensitivity on reading abilities. No significant influence of oculomotor abilities on reading emerged from our analyses, probably due to the sample size and homogeneity. Nevertheless, reading abilities appear to be related to all the components of vision (perceptual, oculomotor, and cognitive) and may benefit from a comprehensive rehabilitation of these aspects from an early age [70,71]. We believe further studies on this topic are necessary, considering bigger samples of patients, attending different school grades and using homogeneous evaluation tools, to better define the learning profiles of children with CVI and reveal whether they could benefit from specific adaptations or training programs, also based on visual functioning. Visual functions and neuropsychological assessments considered in our analyses were performed allowing self-adopted compensation strategies (e.g., head turn, visuo-tactile guidance) and providing appropriate and personalized environmental adaptations depending on specific visual characteristics (e.g., bookrest, room illumination, adequate letters size and line-spacing when testing learning skills). A focus on such environmental adaptations would be recommended in home and school settings and, in general, in the child’s everyday environments. We believe such an approach, in line with current literature [43], would provide more insights on children’s *functional* vision, being worthy of consideration when planning tailored strategies in a multidimensional habilitation approach [70,72] for children with CVI.

Some limitations emerged from the present study. Firstly, its retrospective nature led to some missing data and a reduced homogeneity of evaluations (especially concerning learning abilities), since they were applied for clinical purpose and were necessarily limited by children’s ages and clinical pictures. For example, data on visual field examination could also have proved useful for the analyses and interpretation of results, but a standardized evaluation was unavailable due to the ages and lack of cooperation of the children. Secondly, the inclusion criteria (particularly a verbal IQ > 70) reduced the number of the sample and the width of the spectrum of manifestations, excluding children with a more severe clinical picture and limiting the number of variables to consider. Further research studies are needed that guarantee greater heterogeneity of age and clinical pictures and more homogeneous assessments.

## 5. Conclusions

Our study showed that the visual function profile may contribute to better defining the neuropsychological characteristics of a child with CVI and highlighted the importance of evaluating visual characteristics to defining a functional profile for guiding the habilitation process of children affected by Cerebral Visual Impairment [73].

Among CVI symptoms, particular attention should be given to oculomotor dysfunctions as a cardinal feature of this condition since an early age. Furthermore, a deficit in such visual functions as visual acuity, contrast sensitivity, smooth pursuit, and saccades may negatively affect the development of cognitive visual functions and learning abilities (especially reading skills). Since it has been reported that CVI can negatively affect children’s learning [3], we believe that further studies on this topic might help to shed a light on the possible effects of visual function deficits on reading and computing skills. Finally, our findings showed the necessity of a constant monitoring and the definition of an effective habilitation strategy for visual functions (i.e., perceptual and oculomotor abilities) to sustain the development of *functional* vision and provide children with higher levels of autonomy and inclusion.

## Figures and Tables

**Table 2:** Visual function (perceptual and oculomotor) characteristics of the sample.

Parameter	Category	N (%)
**Near visual acuity**	Normal (>7/10)	32 (63)
	Near-normal (3–7/10)	12 (32)
	Mild low vision (2–3/10)	2 (4)
	Moderate low vision (1–2/10)	1 (2)
	Severe low vision (0.05–1/10)	0 (0)
	Partial blindness (<0.05/10)	0 (0)
	Blindness	0 (0)
	Missing data	4 (8)
**Far visual acuity**	Normal (>7/10)	29 (57)
	Near-normal (3–7/10)	12 (33)
	Mild low vision (2–3/10)	3 (6)
	Moderate low vision (1–2/10)	2 (4)
	Severe low vision (0.05–1/10)	0 (0)
	Partial blindness (<0.05/10)	0 (0)
	Blindness	0 (0)
	Missing data	5 (10)
**Contrast sensitivity**	Normal	33 (65)
	Altered	15 (29)
	Missing data	3 (6)
**Fixation**	Normal (stable, durable, binocular)	23 (45)
	Mildly altered (durable, but alternating or slight difference between the two eyes)	21 (41)
	Slightly instable and/or discontinuous	6 (12)
	Instable and/or discontinuous	0 (0)
	Fluctuating/eccentric	0 (0)
	Occasionally erratic	0 (0)
	Absent response	0 (0)
	Missing data	1 (2)
**Smooth Pursuit**	Durable, complete, and binocular	0 (0)
	Durable but incomplete/asymmetric/non binocular	6 (12)
	Slightly discontinuous in all or great parts of directions	19 (37)
	Clearly discontinuous/augmented latency	22 (43)
	Inconstant/eccentric/fragmented	3 (6)
	Only for small angle	0 (0)
	Absent/no response	0 (0)
	Missing information	1 (2)
**Saccades**	Fluid, complete, normal latency, conjugacy and precision, no evident hypo- or hypermetria	0 (0)
	Fluid but incomplete and/or asymmetric and/or not binocular	4 (8)
	Slight alteration (metria, fluidity, latency)	15 (29)
	Moderate alteration (metria, fluidity, latency)	27 (53)
	Severe alteration/difficult to elicit (metria, fluidity, latency)	4 (8)
	Absent/no response	0 (0)
	Missing information	1 (2)
**Extrinsic ocular motility**	Normal	24 (47)
	Hyperfunction/limitation	19 (37)
	Paralytic limitation	6 (12)
	Missing data	2 (4)

**Table 3:** Cognitive assessment. N indicates the number of subjects who performed the test or had an interpretable result, with the percentage of subjects that completed the test according to age (see Materials and Methods section for abbreviations).

Cognitive Assessment		N (%)
**WPPSI-III**	**VCI**	9 (90)
	**PI**	9 (90)
	**PSI**	4 (40)
	**TIQ**	8 (80)
	**GLI**	3 (30)
**WISC-IV**	**VCI**	36 (87)
	**PRI**	32 (78)
	**WMI**	37 (90)
	**PSI**	35 (85)
	**TIQ**	29 (71)

**Table 4:** Cognitive visual assessment. N indicates the number of subjects who performed the test or had interpretable results.

Visuo-Cognitive Assessment		Category	N
**VMI ^1^**	**VMI ^a^**	normal (>16°p)	18
		frailty (5°–16°p)	4
		deficit (<5°p)	20
		*total*	42
	**VMI-V ^b^**	normal (>16°p)	20
		frailty (5°–16°p)	10
		deficit (<5°p)	11
		*total*	41
	**VMI-M ^c^**	normal (>16°p)	10
		frailty (5°–16°p)	9
		deficit (<5°p)	21
		*total*	40
**DTVP ^2^**	**DTVP-GVP ^a^**	normal (>16°p)	14
		frailty (5–16°p)	6
		deficit (<5°p)	14
		*total*	34
	**DTVP-NMVP ^b^**	normal (>16°p)	15
		frailty (5°–16°p)	14
		deficit (<5°p)	12
		*total*	41
	**DTVP-VMI ^c^**	normal (>16°p)	8
		frailty (5°–16°p)	13
		deficit (<5°p)	13
		*total*	34

**^1^**. Developmental Test of Visual-Motor Integration: (**a**) VMI (global score); (**b**) VMI-V (Visual Perception); (**c**) VMI-M (Motor Coordination) **^2^**. Developmental Test for Visual Perception: (**a**) DTPV-GVP (Developmental Test for Visual Perception—General Visual-Perceptual); (**b**) DTPV-NMVP (Non-Motor Visual-Perceptual); (**c**) DTPV-VMI (Visual-Motor Integration).

**Table 5:** Learning abilities assessment. N indicates the number of subjects who performed the test or had interpretable results.

Leaning Abilities Assessment			Category	N
**DDE-2 ^1^**	**MF ^a^**	**VEL**	normal	18
			deficit	14
			*total*	*32*
		**ERR**	normal	26
			deficit	5
			*total*	*31*
	**NMF ^b^**	**VEL**	normal	12
			deficit	14
			*total*	*26*
		**ERR**	normal	22
			deficit	6
			*total*	*28*
**MT-3 ^2^**	**COMP ^a^**		normal	22
			deficit	13
			*total*	*35*
	**RCOR ^b^**		normal	21
			deficit	5
			*total*	*26*
	**RVEL ^c^**		normal	16
			deficit	12
			*total*	*28*

**^1^**. Battery for Dyslexia and Developmental Dysorthography: (**a**) Meaningful words—Velocity and Error; (**b**) Non Meaningful words—Velocity and Error. **^2^**. MT Test: (**a**) Reading comprehension; (**b**) Reading correctness; (**c**) Reading velocity.

## Data Availability

Data supporting reported results can be found at the link 10.5281/zenodo.5336819 (9 March 2022).

## Supplementary Materials

The following supporting information can be downloaded at: https://www.mdpi.com/article/10.3390/children9060921/s1, Figure S1: Histograms representing the distribution of numerical variables for cognitive tests and the relative p values obtained with Shapiro–Wilk tests. WISC: Wechsler Intelligence Scale for Children. IQ: Intelligence Quotient. VCI: Verbal Comprehension Index. PRI: Perceptual Reasoning Index. WMI: Working Memory Index. PSI: Processing Speed Index; Table S1: Linear regression models for cognitive assessment; Table S2: Ordinal and logistic regression models for cognitive visual and learning abilities as-sessment.
