# Supplementary material for: Visual Function and Neuropsychological Profile in Children with Cerebral Visual Impairment
Source: Children (Basel). 2022 Jun 19;9(6):921. doi: 10.3390/children9060921 (PMC9221908; doi:10.3390/children9060921)
Supplement: Supplementary file 1 [file children-09-00921-s001.zip › children-1697681-supplementary.pdf]

children

MDPI

1. Variables Distribution

We report the histogram representation of the distribution of the numerical variables concerning cognitive tests and the results of normality tests. Each histogram represents the distribution of a variable (WISC-IQ, WISC-VCI, WISC-PRI, WISC-WMI, WISC-PSI). Shapiro–Wilk tests do not show evidence of non-normality as deduced by the reported p values.

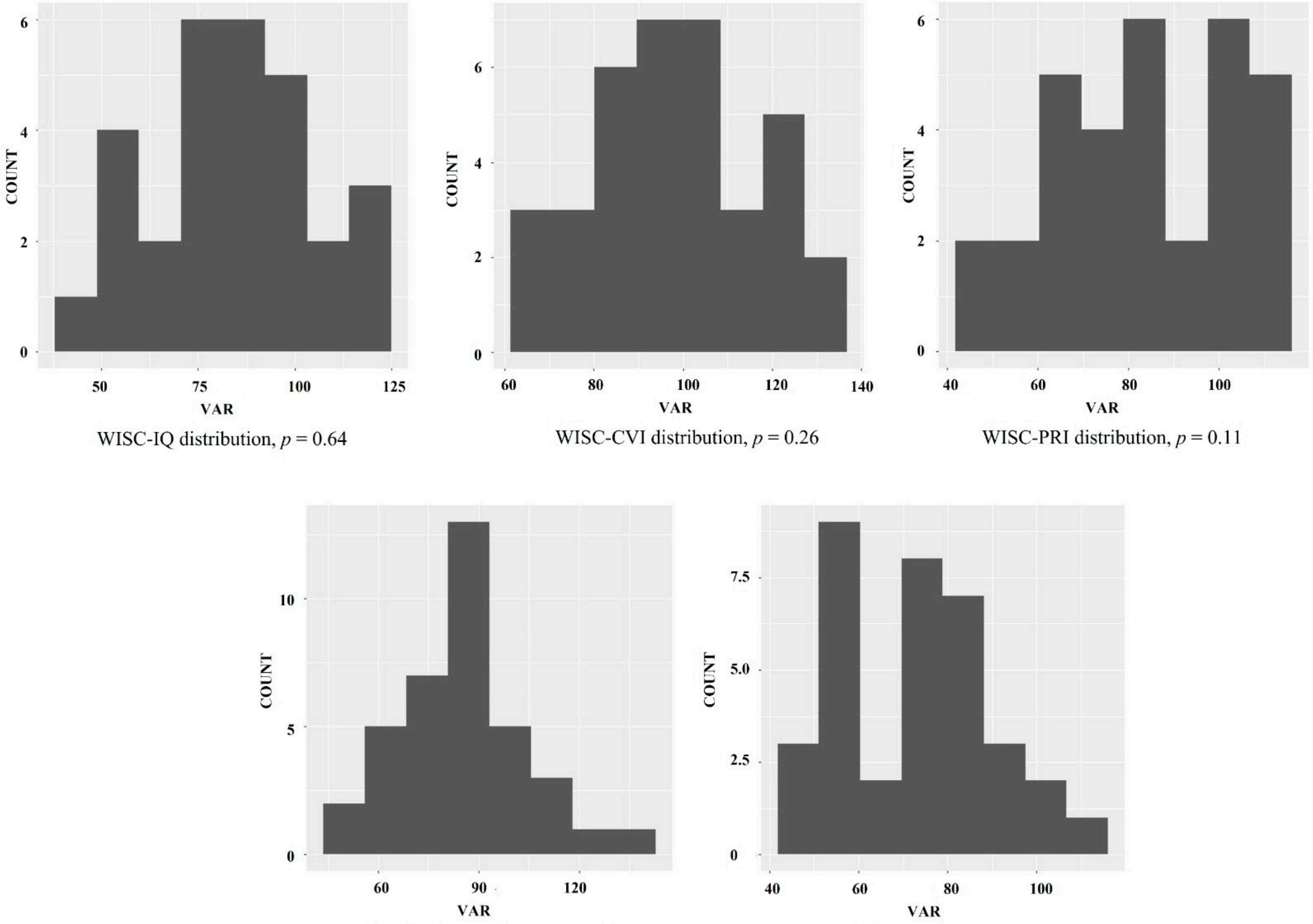

WISC-IQ distribution, $p = 0.64$ WISC-CVI distribution, $p = 0.26$ WISC-PRI distribution, $p = 0.11$

WISC-WMI distribution, $p = 0.75$ WISC-PSI distribution, $p = 0.75$

Figure S1. Histograms representing the distribution of numerical variables for cognitive tests and the relative p values obtained with Shapiro–Wilk tests. WISC: Wechsler Intelligence Scale for Children. IQ: Intelligence Quotient. VCI: Verbal Comprehension Index. PRI: Perceptual Reasoning Index. WMI: Working Memory Index. PSI: Processing Speed Index.

2. Supplementary Results

For each target variable, an appropriate parametric regression model is built with the following variables as covariates: Neurological Signs, Best Corrected Visual Acuity (for near), Best Corrected Visual Acuity (for distance), Fixation, Smooth Pursuit, Saccades, Ocular Motility, Contrast Sensitivity.

Under the target variable, the global significance of the model is reported.

Table S1. Linear regression models for cognitive assessment.

	Coefficient	Standard Error	*t* Value	*p* Value
Neurological Signs	−2.0036	3.0100	−0.666	0.512

WISC-VCI (Verbal Comprehension Index) overall significance p = 0.133	Best Corrected Visual Acuity (for near)	3.4535	4.4744	0.772	0.447
	Best Corrected Visual Acuity (for distance)	0.8335	3.6729	0.227	0.822
	Fixation	9.7140	6.1128	1.589	0.125
	Smooth Pursuit	−13.6268	8.3445	−1.633	0.115
	Saccades	−1.5589	7.8697	−0.198	0.845
	Ocular Motility	−5.6862	5.5031	−1.033	0.311
	Contrast Sensitivity	−11.2218	6.3845	−1.758	0.091
WISC-PRI (Perceptual Reasoning Index) overall significance p = 0.014	Neurological Signs	3.301	3.195	1.033	0.3133
	Best Corrected Visual Acuity (for near)	6.300	5.144	1.225	0.2342
	Best Corrected Visual Acuity (for distance)	−15.106	4.106	−3.679	0.0014 **
	Fixation	2.388	8.566	0.279	0.7832
	Smooth Pursuit	−16.416	9.297	−1.766	0.0920 .
	Saccades	−8.715	8.345	−1.044	0.3082
	Ocular Motility	3.138	6.441	0.487	0.6312
	Contrast Sensitivity	−14.283	7.342	−1.945	0.0652
WISC-WMI (Working Memory Index) overall significance p = 0.039	Neurological Signs	2.593	3.758	0.690	0.4961
	Best Corrected Visual Acuity (for near)	8.880	5.695	1.559	0.1306
	Best Corrected Visual Acuity (for distance)	−9.122	4.584	−1.990	0.0568
	Fixation	−1.542	7.485	−0.206	0.8383
	Smooth Pursuit	−18.122	10.516	−1.723	0.0963
	Saccades	−3.138	9.636	−0.326	0.7472
	Ocular Motility	−4.557	6.981	−0.653	0.5194
	Contrast Sensitivity	−20.609	7.876	−2.616	0.0144*
WISC-PSI (Processing Speed Index) overall significance p = 0.123	Neurological Signs	3.732	2.931	1.273	0.21512
	Best Corrected Visual Acuity (for near)	9.480	4.607	2.058	0.05064
	Best Corrected Visual Acuity (for distance)	−11.032	3.754	−2.939	0.00717**
	Fixation	−14.022	7.211	−1.945	0.06364
	Smooth Pursuit	−19.419	8.164	−2.379	0.02568 *
	Saccades	6.900	8.294	0.832	0.41366
	Ocular Motility	1.874	5.970	0.314	0.75630
	Contrast Sensitivity	−4.371	6.510	−0.671	0.50843
WISC-IQ (Intelligence Quotient) overall significance p = 0.301	Neurological Signs	2.5476	4.3097	0.591	0.5618
	Best Corrected Visual Acuity (for near)	10.3161	7.0759	1.458	0.1621
	Best Corrected Visual Acuity (for distance)	−10.9858	5.6331	−1.950	0.0669
	Fixation	−5.2629	11.0749	−0.475	0.6404
	Smooth Pursuit	−20.2035	13.0731	−1.545	0.1396
	Saccades	−2.5183	12.4570	−0.202	0.8421
	Ocular Motility	−0.8547	9.0261	−0.095	0.9256
	Contrast Sensitivity	−22.5585	10.2020	−2.211	0.0402 *

* $p < 0.05$; ** $p < 0.0.1$

Table S2. Ordinal and logistic regression models for cognitive visual and learning abilities assessment.

		Coefficient	Standard Error	*t* Value	*p* Value
VMI (Developmental Test of Visual-Motor Integration) overall significance p = 0.026	Best Corrected Visual Acuity (for near)	−2.0062	0.9543	−2.1023	0.0355 *
	Best Corrected Visual Acuity (for distance)	2.7518	1.3224	2.0809	0.0374 *
	Fixation	2.3153	1.9112	1.2114	0.2257
	Smooth Pursuit	4.2792	2.1423	1.9975	0.0458 *
	Saccades	−1.6836	1.6188	−1.0401	0.2983
	Ocular Motility	−0.4568	1.8860	−0.2422	0.8086
	Contrast Sensitivity	1.5055	1.3745	1.0953	0.2734
VMI-V (Visual Perception) overall significance p < 0.001	Best Corrected Visual Acuity (for near)	−2.7694	1.3455	−2.0582	0.0396 *
	Best Corrected Visual Acuity (for distance)	6.1976	2.2755	2.7236	0.0065 *
	Fixation	3.1211	1.9085	1.6354	0.1020
	Smooth Pursuit	6.7864	2.6342	2.5763	0.0100 *
	Saccades	−1.6454	1.7261	−0.9533	0.3405
	Ocular Motility	0.4234	1.4335	0.2953	0.7677
	Contrast Sensitivity	3.0431	1.7766	1.7129	0.0867
VMI-M (Motor Coordination) overall significance p = 0.007	Best Corrected Visual Acuity (for near)	0.4579	1.3199	0.3470	0.7286
	Best Corrected Visual Acuity (for distance)	0.8223	1.6133	0.5097	0.6103
	Fixation	1.8018	2.4069	0.7486	0.4541
	Smooth Pursuit	4.5275	2.1086	2.1472	0.0318 *
	Saccades	−3.1166	1.7023	−1.8308	0.0671
	Ocular Motility	0.0538	2.3994	0.0224	0.9821
	Contrast Sensitivity	0.8697	1.3432	0.6475	0.5173
DTPV-GVP (Developmental Test for Visual Perception-General Visual-Perceptual) overall significance p = 0.157	Best Corrected Visual Acuity (for near)	−0.6271	0.6678	−0.9391	0.3477
	Best Corrected Visual Acuity (for distance)	1.7207	0.9336	1.8431	0.0653
	Fixation	1.7541	1.3755	1.2753	0.2022
	Smooth Pursuit	1.7977	1.6784	1.0711	0.2841
	Saccades	−0.3167	1.7684	−0.1791	0.8579
	Ocular Motility	0.0041	1.1579	0.0036	0.9972
	Contrast Sensitivity	1.1336	1.0395	1.0906	0.2755

DTPV-NMVP (Non-Motor Visual-Perceptual) ***overall significance p = 0.238***	Best Corrected Visual Acuity (for near)	0.1251	0.5365	0.2333	0.8156
	Best Corrected Visual Acuity (for distance)	1.2103	0.7367	1.6428	0.1004
	Fixation	0.3315	0.8818	0.3759	0.7070
	Smooth Pursuit	0.7177	1.3354	0.5375	0.5909
	Saccades	0.4423	1.3786	0.3209	0.7483
	Ocular Motility	−0.0164	0.8252	−0.0198	0.9842
	Contrast Sensitivity	0.5775	0.8645	0.6681	0.5041
DTPV-VMI (Visual-Motor Integration) ***overall significance p = 0.096***	Best Corrected Visual Acuity (for near)	0.0897	0.4623	0.1940	0.8462
	Best Corrected Visual Acuity (for distance)	−0.7498	0.4349	−1.7240	0.0847
	Fixation	−0.7824	0.7139	−1.0959	0.2731
	Smooth Pursuit	1.4492	1.0111	1.4333	0.1518
	Saccades	−2.0781	1.0572	−1.9657	0.0493*
	Ocular Motility	1.5491	0.6929	2.2355	0.0254*
	Contrast Sensitivity	0.3354	0.7141	0.4697	0.6385
DDE-MF-VEL (Battery for Dyslexia and Developmental Dysorthography – Meaningful - Speed) overall significance p = 0.312	Best Corrected Visual Acuity (for near)	−1.1329	1.0549	−1.074	0.2829
	Best Corrected Visual Acuity (for distance)	1.2037	1.1222	1.073	0.2834
	Fixation	1.1852	1.4829	0.799	0.4242
	Smooth Pursuit	2.2146	1.5272	1.450	0.1470
	Saccades	−2.9326	1.9064	−1.538	0.1240
	Ocular Motility	1.0753	1.4423	0.746	0.4559
	Contrast Sensitivity	2.1349	1.2322	1.733	0.0832
DDE-MF-ERR (Battery for Dyslexia and Developmental Dysorthography – Meaningful - Error) overall significance p = 0.160	Best Corrected Visual Acuity (for near)	−1.2851	1.7830	−0.721	0.471
	Best Corrected Visual Acuity (for distance)	0.1227	1.3767	0.089	0.929
	Fixation	1.2909	2.6649	0.484	0.628
	Smooth Pursuit	26.0830	4385.6549	0.006	0.995
	Saccades	−1.5850	3.3259	−0.477	0.634
	Ocular Motility	2.4737	3.0477	0.812	0.417
	Contrast Sensitivity	25.9807	4385.6542	0.006	0.995
DDE-NMF-VEL	Best Corrected Visual Acuity (for near)	−37.327	10678.207	−0.003	0.997

(Battery for Dyslexia and Developmental Dysorthography – Non-Meaningful - Speed) overall significance p = 0.008	Best Corrected Visual Acuity (for distance)	82.100	31301.314	0.003	0.998
	Fixation	113.867	27318.983	0.004	0.997
	Smooth Pursuit	150.806	36993.178	0.004	0.997
	Saccades	−113.089	27318.983	−0.004	0.997
	Ocular Motility	−5.227	41614.035	0.000	1.000
	Contrast Sensitivity	82.879	31301.313	0.003	0.998
DDE-NMF-ERR (Battery for Dyslexia and Developmental Dysorthography – Non-Meaningful - Error) overall significance p = 0.666	Best Corrected Visual Acuity (for near)	1.26771	0.90158	1.406	0.160
	Best Corrected Visual Acuity (for distance)	0.55833	0.87765	0.636	0.525
	Fixation	−1.72075	1.54763	−1.112	0.266
	Smooth Pursuit	−0.54766	1.62030	−0.338	0.735
	Saccades	1.86936	1.83030	1.021	0.307
	Ocular Motility	1.42975	1.73111	0.826	0.409
	Contrast Sensitivity	1.14796	1.84243	0.623	0.533
MT-RVEL (MT Test – Reading speed) overall significance p < 0.001	Best Corrected Visual Acuity (for near)	−29.275	78419.814	0.000	1.000
	Best Corrected Visual Acuity (for distance)	−11.066	55025.391	0.000	1.000
	Fixation	8.397	91362.532	0.000	1.000
	Smooth Pursuit	54.039	406658.409	0.000	1.000
	Saccades	13.598	284757.764	0.000	1.000
	Ocular Motility	−1.052	147221.439	0.000	1.000
	Contrast Sensitivity	75.665	122824.854	0.001	1.000
**MT-RCOR (MT Test – Reading correctness)** ***overall significance p = 0.247***	Best Corrected Visual Acuity (for near)	−3.557	4.917	−0.723	0.469
	Best Corrected Visual Acuity (for distance)	−1.739	2.673	−0.651	0.515
	Fixation	3.019	5.156	0.586	0.558
	Smooth Pursuit	4.383	5.218	0.840	0.401
	Saccades	−5.260	5.340	−0.985	0.325
	Ocular Motility	4.914	4.571	1.075	0.282
	Contrast Sensitivity	2.931	4.847	0.605	0.545
**MT-COMP**	Best Corrected Visual Acuity (for near)	−0.5644	0.9192	−0.614	0.53920

**(MT Test – Reading comprehension)** ***overall significance p = 0.049***	Best Corrected Visual Acuity (for distance)	0.2237	0.8161	0.274	0.78397
	Fixation	0.2872	0.9997	0.287	0.77389
	Smooth Pursuit	2.6014	2.2321	1.165	0.24383
	Saccades	−2.6630	1.9760	−1.348	0.17776
	Ocular Motility	1.9569	1.3218	1.480	0.13876
	Contrast Sensitivity	4.3645	1.6303	2.677	0.00743**

* $p < 0.05$; ** $p < 0.01$.
